# Structure-function specialisation of the interfascicular matrix in the human achilles tendon

**DOI:** 10.1016/j.actbio.2021.07.019

**Published:** 2021-09-01

**Authors:** Dharmesh Patel, Danae E. Zamboulis, Ewa M. Spiesz, Helen L. Birch, Peter D. Clegg, Chavaunne T. Thorpe, Hazel R.C. Screen

**Affiliations:** a Institute of Bioengineering, School of Engineering and Materials Science, Queen Mary University of London, Mile End Road, London E1 4NS, UK; b Department of Musculoskeletal and Ageing Science, Institute of Life Course and Medical Sciences, University of Liverpool, West Derby Street, Liverpool L7 8TX, UK; c Department of Bionanoscience, Delft University of Technology, Building 58, Van der Maasweg 9, 2629 HZ Delft, the Netherlands; d Institute of Orthopaedics and Musculoskeletal Science, UCL, Stanmore Campus, Brockley Hill, Stanmore, Middlesex, HA7 4LP, UK; e The Medical Research Council Versus Arthritis Centre for Integrated Research into Musculoskeletal Ageing (CIMA), University of Liverpool, West Derby Street, Liverpool L7 8TX, UK; f Department of Comparative Biomedical Sciences, Royal Veterinary College, Royal College Street, London NW1 0TU, UK

**Keywords:** Anterior tibialis tendon, Fascicles, Interfascicular matrix, Mechanical testing, Mass spectrometry, Ageing

## Abstract

Tendon consists of highly aligned collagen-rich fascicles surrounded by interfascicular matrix (IFM). Some tendons act as energy stores to improve locomotion efficiency, but such tendons commonly obtain debilitating injuries. In equine tendons, energy storing is achieved primarily through specialisation of the IFM. However, no studies have investigated IFM structure-function specialisation in human tendons. Here, we compare the human positional anterior tibial tendon and energy storing Achilles tendons, testing the hypothesis that the Achilles tendon IFM has specialised composition and mechanical properties, which are lost with ageing. Data demonstrate IFM specialisation in the energy storing Achilles, with greater elasticity and fatigue resistance than in the positional anterior tibial tendon. With ageing, alterations occur predominantly to the proteome of the Achilles IFM, which are likely responsible for the observed trends towards decreased fatigue resistance. Knowledge of these key energy storing specialisations and their changes with ageing offers crucial insight towards developing treatments for tendinopathy.

**Statement of significance:**

Developing effective therapeutics or preventative measures for tendon injury necessitates the understanding of healthy tendon function and mechanics. By establishing structure-function relationships in human tendon and determining how these are affected by ageing, potential targets for therapeutics can be identified. In this study, we have used a combination of mechanical testing, immunolabelling and proteomics analysis to study structure-function specialisations in human tendon. We demonstrate that the interfascicular matrix is specialised for energy storing in the Achilles tendon, and that its proteome is altered with ageing, which is likely responsible for the observed trends towards decreased fatigue resistance. Knowledge of these key energy storing specialisations and their changes with ageing offers crucial insight towards developing treatments and preventative approaches for tendinopathy.

## Graphical abstract

**Figure:**
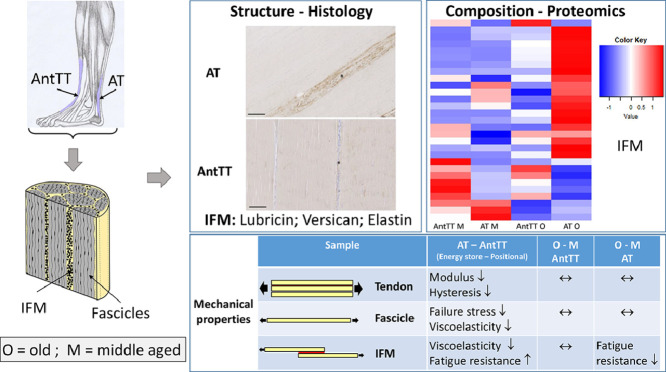

## 1 Introduction

The human Achilles tendon has two functions; transferring muscle-generated forces to move the skeleton, and storing energy during locomotion. Efficient energy storage requires the ability to stretch and recoil with each stride, in contrast to positional tendons such as the anterior tibial tendon, which are relatively stiff to enable efficient force transfer (reviewed by [1,2]). It is well established that the mechanical properties of the Achilles tendon are specialised to increase locomotory efficiency and allow the tendon to resist the high stresses and strains experienced during use [3,4]. Despite these specialisations, the Achilles tendon has a much lower safety factor than the positional anterior tibial tendon [5,6], making it prone to age-related tendinopathy [7], [8], [9]. While fundamental Achilles tendon structure is well defined, the structural specialisations that provide the human Achilles tendon with the mechanical properties required for energy storage remain unclear. This information is crucial to develop novel, targeted treatments that recover Achilles function post-injury.

All tendons have the same general structure, comprised of aligned, collagen-rich fascicles bound by the interfascicular matrix (IFM); a looser, less organised matrix also referred to as the endotenon [10,11]. Our previous studies using the equine model have identified the importance of the IFM for tendon function, with structural and compositional specialisations within the IFM of the energy storing superficial digital flexor tendon (SDFT) resulting in a highly extensible and fatigue resistant material which likely translates to greater extensibility and fatigue resistance within the whole tendon [12], [13], [14], [15], [16]. However, these specialisations become compromised with ageing, likely contributing to the age-related increased risk of injury to energy storing tendons in horses [14,17,18]. Studies have also identified differences in the collagenous components of energy storing and positional tendons, with fascicles from energy storing tendons having a helical structure which provides enhanced fatigue resistance [19], and differences in crosslinking between tendon types limiting structural disruption in response to overload in fibrils from energy storing tendons [20].

While structure-function relationships in equine, and other mammalian tendons are now well established, little work has been undertaken to elucidate structure-function relationships in human tendon or how these alter with ageing. Indeed, to the authors’ knowledge, only one study has directly compared functionally distinct human tendons, demonstrating that the Achilles has a lower elastic modulus than the anterior tibial tendon, which is accompanied by several compositional differences [1]. While studies have assessed the mechanical properties of the Achilles tendon *in vivo* [3,21], or obtained healthy and/or diseased Achilles tissue via biopsy or during surgery [22], [23], [24], the limited amount of tissue available with these approaches restricts the subsequent analysis that can be performed. Further, no studies have compared subunits of functionally distinct human tendons to establish their role in tendon structure-function relationships.

The aim of this study was therefore to compare the composition and mechanical properties of the human Achilles and anterior tibial tendons and their subunits, and identify any age-related alterations. We hypothesise that the IFM in the energy storing Achilles tendon has specialised composition and mechanical properties, and these specialisations are lost with ageing.

## 2 Materials & methods

### 2.1 Sample collection and processing

Collection, storage and use of human tendon was approved by the Human Tissue Authority (HTA; REC number: 14/NE/0154). Achilles and anterior tibial tendons were collected, either post-mortem from the Centre for Comparative and Clinical Anatomy, University of Bristol (HTA licence: 12135), or the Newcastle Surgical Training Centre, Freeman Hospital, Newcastle upon Tyne (HTA licence: 12148), or as surgical waste from limbs amputated for tumour treatment at the Royal National Orthopaedic Hospital, Stanmore (UCL/UCLH Biobank for Studying Health and Disease; HTA license: 12055; R&D approval from UCL/UCLH/RF JRO (Ref: 11/0464)). Donors were divided into two age groups (n=9/group); middle-aged: 31–58 years, mean 47.6 years (3 female, 6 male); old aged: 72–94 years, mean 84.8 years (4 female, 5 male). Paired Achilles and anterior tibial tendons were processed <24 hours post-mortem by quartering longitudinally, and either frozen for mechanical analysis or prepared for proteomic and histological analysis as described in supplementary methods [14,25].

All donor tendons underwent mechanical characterisation of IFM and fascicles. Owing to the challenges associated with gripping short specimens for mechanical testing, and time required to laser-capture tissue for proteomic analysis, only 5 paired tendons from each age group underwent quarter tendon testing, histologic and proteomic analysis. See Supplementary Table 1 for donor and analysis details.

### 2.2 Mechanical characterisation of tendon, fascicles and IFM

#### 2.2.1 Tendon quasi-static mechanical properties

Immediately before testing, one quarter from each tendon was thawed and its cross-sectional area measured [26]. Samples were preconditioned then pulled to failure. See supplementary methods for details of testing parameters.

#### 2.2.2 Fascicle and IFM quasi-static mechanical properties

Samples for determination of fascicle and IFM quasi-static properties were dissected and prepared from one of the tendon quarters as previously described [12,27]. Samples were preconditioned then pulled to failure. See supplementary methods for details of dissection and testing parameters.

#### 2.2.3 Calculation of viscoelastic and failure properties

Force and extension data were recorded at 100Hz. Hysteresis and stress relaxation were calculated during preconditioning. For tendons and fascicles, maximum modulus, failure strain and stress were calculated during the pull-to-failure test, and for IFM samples, maximum stiffness, and force and extension at yield and failure were calculated as previously described [13].

#### 2.2.4 Fascicle and IFM fatigue properties

Samples for determination of fascicle and IFM fatigue properties were dissected from one of the tendon quarters and fatigue properties measured by subjecting samples to cyclic loading until failure as described previously [15,28], with some modifications. See supplementary methods for details of testing parameters. Force and displacement data were collected at 100Hz, with cycles to failure and secondary creep rate calculated for each sample.

#### 2.2.5 Statistical analysis

A general linear mixed model was applied to assess differences between tendon type and with ageing, using tendon type and age as crossed factors and donor as a nested random effect factor (R, v3.6.1; www.r-project.org). Shapiro-Wilk tests indicated non-normal distribution of the data, hence data transformations using Box-Cox transformations were used.

### 2.3 Protein immunolocalisation

Immunohistochemistry was used to localise decorin, fibromodulin, lubricin and versican, as described previously [25]. See Supplementary Table 2 for antibodies and blocking conditions. Elastin distribution was assessed by elastic van Gieson's staining.

### 2.4 Quantification of elastin content

The elastin content of Achilles and anterior tibial tendons from middle-aged and old donors (n=3/group) was quantified using the FASTIN^TM^ Elastin Assay (Biocolor, UK) as previously described [16], using two technical replicates per sample (Coefficient of variation: range 0.17 - 10.39%; mean 4.52%). Differences between tendon types assessed using paired t-tests.

### 2.5 Mass spectrometry analyses

#### 2.5.1 Laser-capture microdissection

Laser-capture microdissection of IFM and fascicles from tendon cryosections was performed as previously described [14], with a haematoxylin staining step (1 min.) to visualise cell nuclei.

#### 2.5.2 Protein Extraction and mass spectrometry analyses

Protein extraction and mass spectrometry analysis of laser-captured samples was performed as described previously [14], using an Ultimate 3000 Nano system (Dionex/Thermo Fisher Scientific) coupled to a Q-Exactive Quadrupole-Orbitrap mass spectrometer (Thermo-Scientific).

#### 2.5.3 Peptide identification and quantification

Fascicle and IFM proteins were identified, searching against the UniHuman reviewed database (http://www.uniprot.org/proteomes/) using parameters and filters as described previously [29] (Peaks® 8.5 PTM software; Bioinformatics Solutions, Canada). Label-free quantification was performed separately for IFM and fascicles from each tendon and age group (Peaks® 8.5 PTM software). Protein abundances were normalised for collected laser-capture area and differentially abundant proteins between groups identified using fold change ≥2 and *p*<0.05 (PEAKS-adjusted p-values).The mass spectrometry data have been deposited to the ProteomeXchange Consortium (http://proteomecentral.proteomexchange.org) via the PRIDE partner repository [30] with the dataset identifier PXD018212.

#### 2.5.4 Gene ontology and network analysis

The dataset of differentially abundant proteins between groups were classified for cell compartment association with Ingenuity Pathway Analysis (IPA, Qiagen) and for matrisome categories using The Matrisome Project database [31]. Protein pathway analysis for differentially abundant proteins was performed in IPA.

## 3 Results

### 3.1 Tendon, fascicle and IFM mechanical properties

**Fig. 1:**
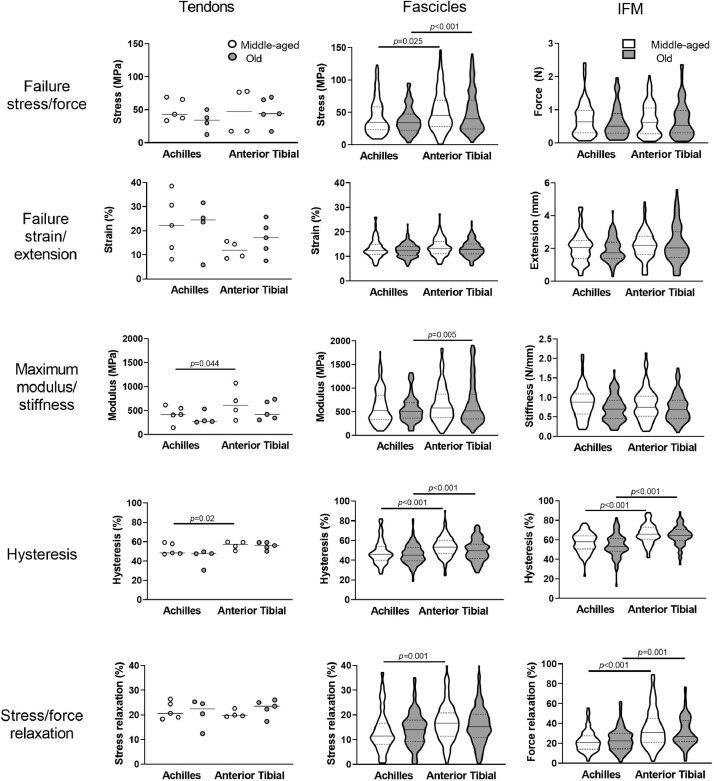
Failure and viscoelastic properties of Achilles and anterior tibial tendons and their subunits from middle-aged and old donors. Due to limited sample numbers, individual data points are plotted for tendon tests (solid line denotes median). Distribution of fascicle and IFM data is shown by violin plots (solid line denotes median, dashed lines indicate the interquartile range and width corresponds to frequency of data points).

There were no differences in tendon mechanical properties between age groups or tendon types, except for maximum modulus and hysteresis, which were significantly greater in the anterior tibial tendon (Fig. 1). However, notable variability between donors was evident. At the fascicle level, ultimate tensile stress, hysteresis and stress relaxation were significantly greater in fascicles from the anterior tibial tendon compared to those from the Achilles, however, no changes in fascicle mechanical properties were evident with ageing. Similarly, significantly greater hysteresis and force relaxation were evident in the IFM of the anterior tibial tendon compared to the Achilles, but no changes occurred with ageing. There were also no differences in IFM extension or force at the yield point, defined as the point at which maximum stiffness was reached and indicating the limit of elastic behaviour, between tendon types or age groups (Supplementary Fig. 1).

### 3.2 Fascicle and IFM fatigue properties

**Fig. 2:**
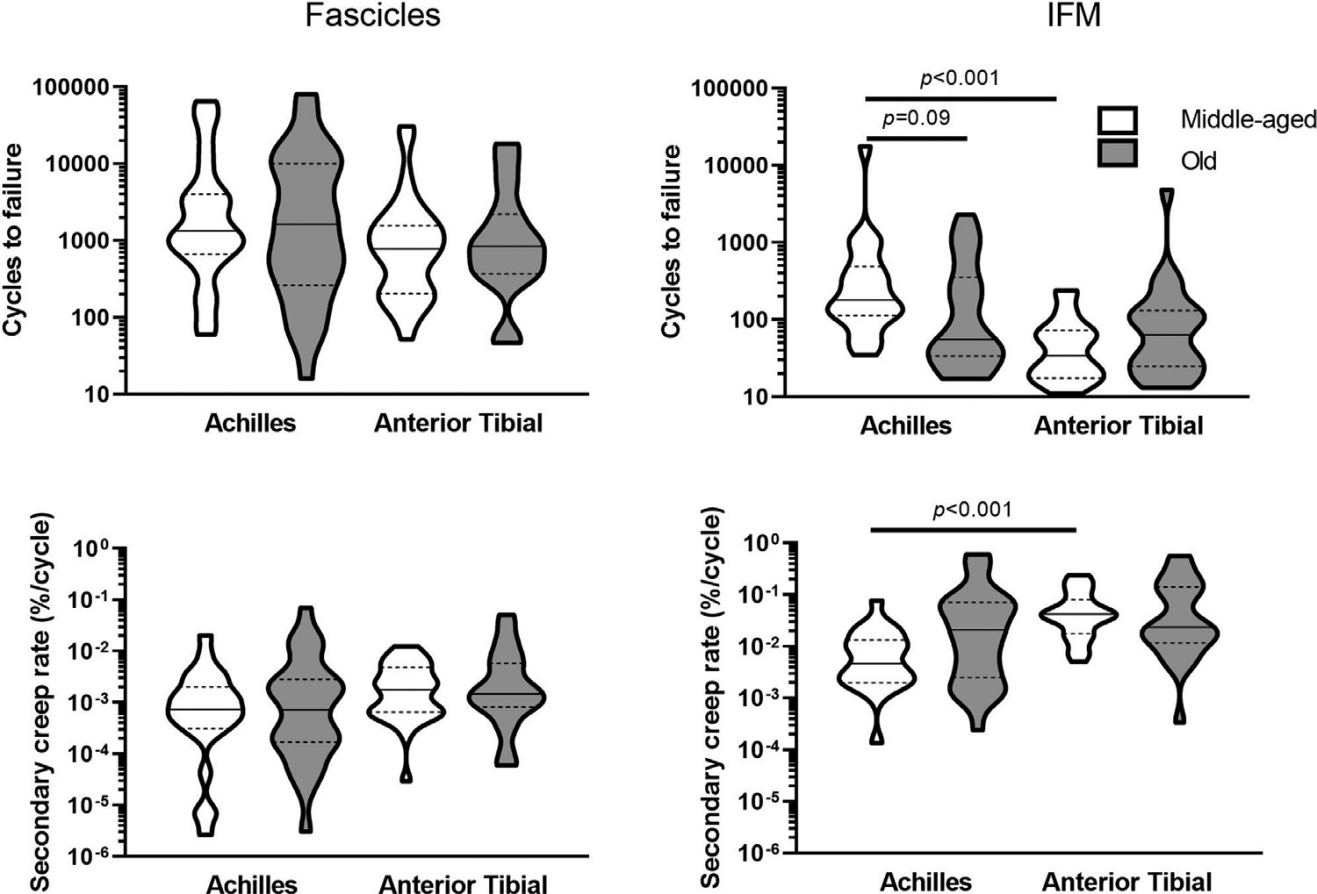
Fatigue properties of fascicles and IFM in Achilles and anterior tibial tendons. Data are plotted on a log_10_ scale. Distribution of data is shown by violin plots (solid line denotes median, dashed lines indicate the interquartile range and width corresponds to frequency of data points).

Fascicle fatigue resistance did not differ between tendon types or with ageing. By contrast, the IFM in the Achilles was more fatigue resistant than that in the anterior tibial tendon, characterised by a significantly greater number of cycles to failure (p<0.001) and lower rate of secondary creep (p<0.001). These differences were lost with ageing, due to indications of reduced fatigue resistance in the ageing Achilles IFM, seen in a trend towards a decrease in number of cycles to failure (p=0.09; Fig. 2).

### 3.3 Protein immunolocalisation

**Fig. 3:**
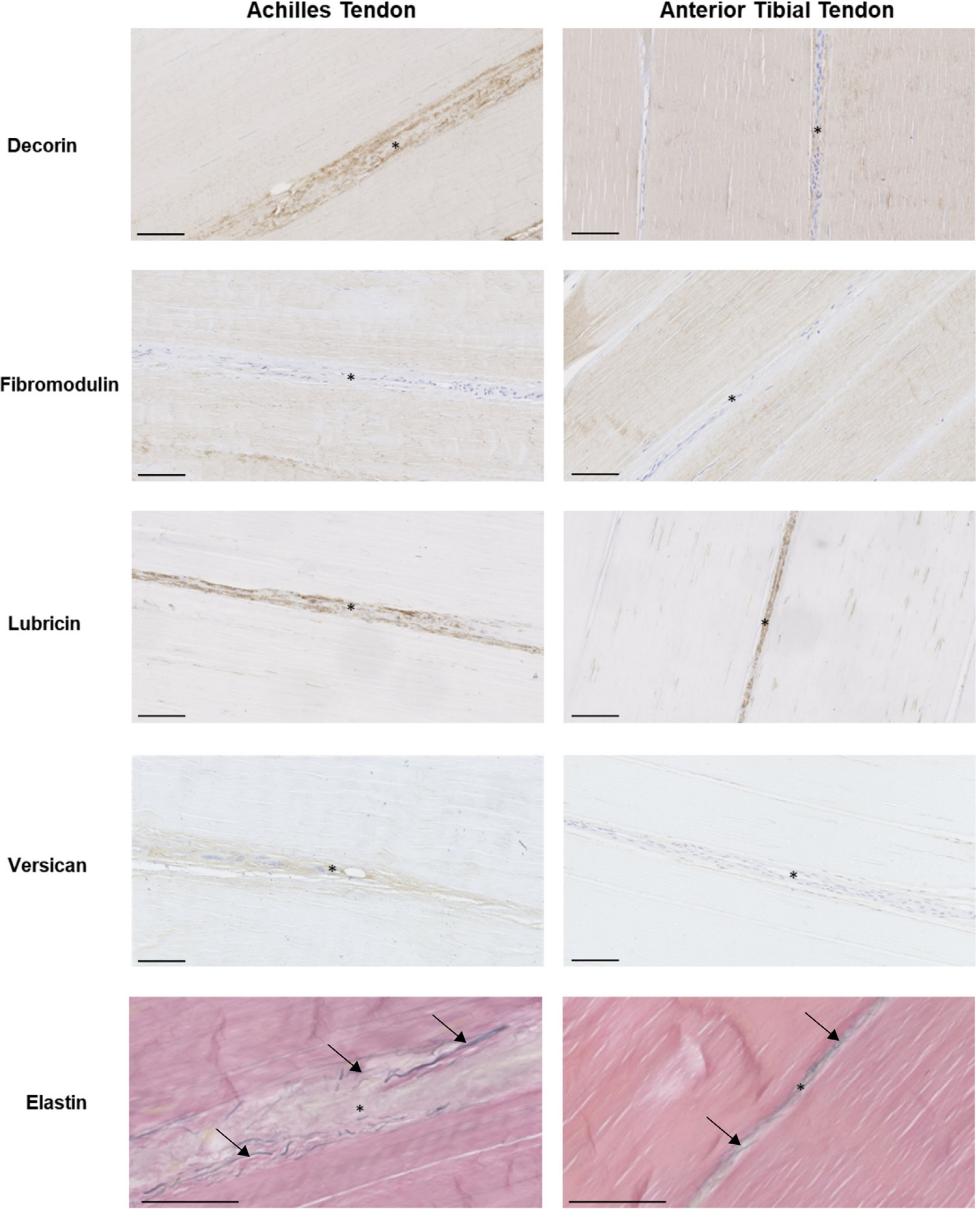
Localisation of proteins in the Achilles and anterior tibial tendons. Representative immunohistochemical images showing distribution of tendon proteoglycans (brown) and elastin (black; indicated by arrows) in the Achilles and anterior tibialis tendons from middle-aged donors. IFM is indicated by *. Scale bar = 100 µm.

Lubricin, versican and elastin localised to the IFM in both tendon types. Fibromodulin staining was greater in the fascicles, with little or no staining in the IFM. Decorin was present throughout the extracellular matrix (ECM) in both tendon types (Fig. 3).

### 3.4 Tendon elastin content

Elastin content in the Achilles from middle-aged and old donors was 2.4±1.6% and 1.05±0.4% respectively. Elastin content in the anterior tibial was 1.7±0.9% in middle-aged, and 2.1±0.6% in old tendon. See Supplementary Fig. 2 for individual data. There was a trend towards a lower elastin content in the old Achilles compared to the old anterior tibial tendon, but this did not reach statistical significance (p=0.07).

### 3.5 Protein identification and ontology

Overall, more proteins were identified in the IFM than in fascicles, and a greater proportion of those identified in fascicles were classified as ECM or ECM-related proteins (Table 1).

**Table 1:** Protein number in each of the tendon compartments as identified by LC-MS/MS and categorisation of matrisome-associated proteins. Numbers in brackets indicate percentage of total protein number.

	Fascicular Matrix				Interfascicular Matrix			
	Achilles		Anterior tibial		Achilles		Anterior tibial	
	Middle	Old	Middle	Old	Middle	Old	Middle	Old
Total Protein Number	152	152	148	153	265	259	243	211
Number of ECM Proteins	54 (35.5%)	69 (45.4%)	58 (39.2%)	53 (34.6%)	77 (29.1%)	81 (31.3%)	82 (33.7%)	74 (35.1%)
ECM Glycoproteins	16 (10.5%)	22 (14.5%)	17 (11.5%)	16 (10.5%)	28 (10.6%)	31 (12.0%)	31 (12.8%)	29 (13.7%)
Collagens	15 (9.9%)	16 (10.5%)	15 (10.1%)	15 (9.8%)	20 (7.5%)	18 (6.9%)	17 (7.0%)	17 (8.1%)
Proteoglycans	9 (5.9%)	10 (6.6%)	9 (6.1%)	8 (5.2%)	11 (4.2%)	11 (4.2%)	10 (4.1%)	10 (4.7%)
ECM Affiliated Proteins	4 (2.6%)	6 (3.9%)	6 (4.1%)	5 (3.3%)	9 (3.4%)	8 (3.1%)	9 (3.7%)	7 (3.3%)
ECM Regulators	8 (5.3%)	13 (8.6%)	10 (6.8%)	8 (5.2%)	6 (2.3%)	11 (4.2%)	13 (5.3%)	9 (4.3%)
Secreted Factors	2 (1.3%)	2 (1.3%)	1 (0.7%)	1 (0.7%)	3 (1.1%)	2 (0.8%)	2 (0.8%)	2 (0.9%)

#### 3.5.1 Differences in protein abundance between tendon types and age groups

**Fig. 4:**
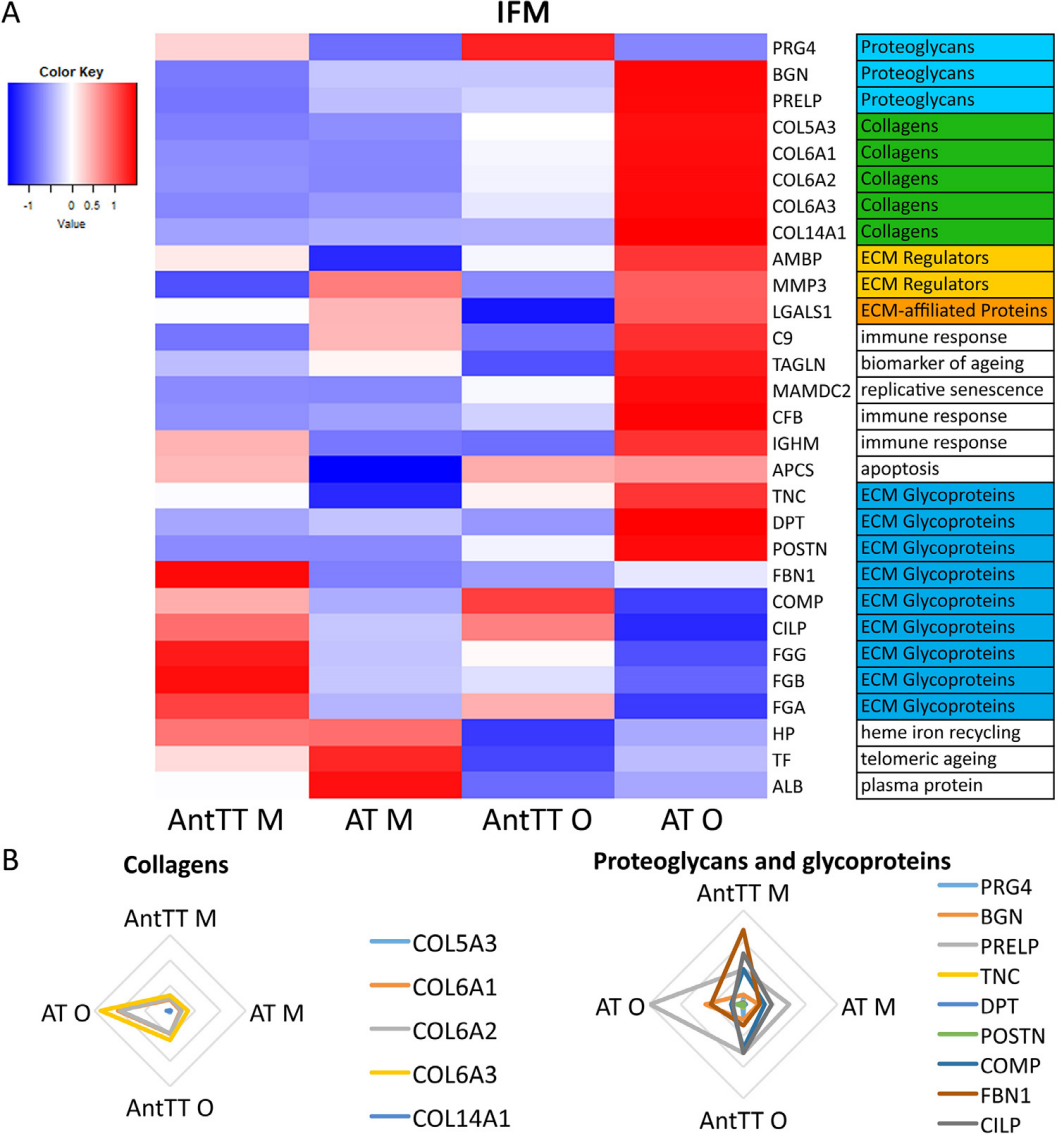
Most changes in the IFM protein abundance are observed in the old Achilles tendon. (A) Heatmap of differentially abundant proteins in the IFM middle-aged anterior tibial (AntTT M) and Achilles tendon (AT M), and old anterior tibialis tendon (AntTT O) and Achilles tendon (AT O) (p<0.05, fold change ≥2). Heatmap colour scale ranges from blue to white to red with blue representing lower abundance and red higher abundance. MatrisomeDB was used to assign protein classifications. (B) Radar plots of collagens, proteoglycans and glycoproteins that showed differential abundance with age or tendon type in the IFM (p<0.05, fold change ≥2).

**Fig. 5:**
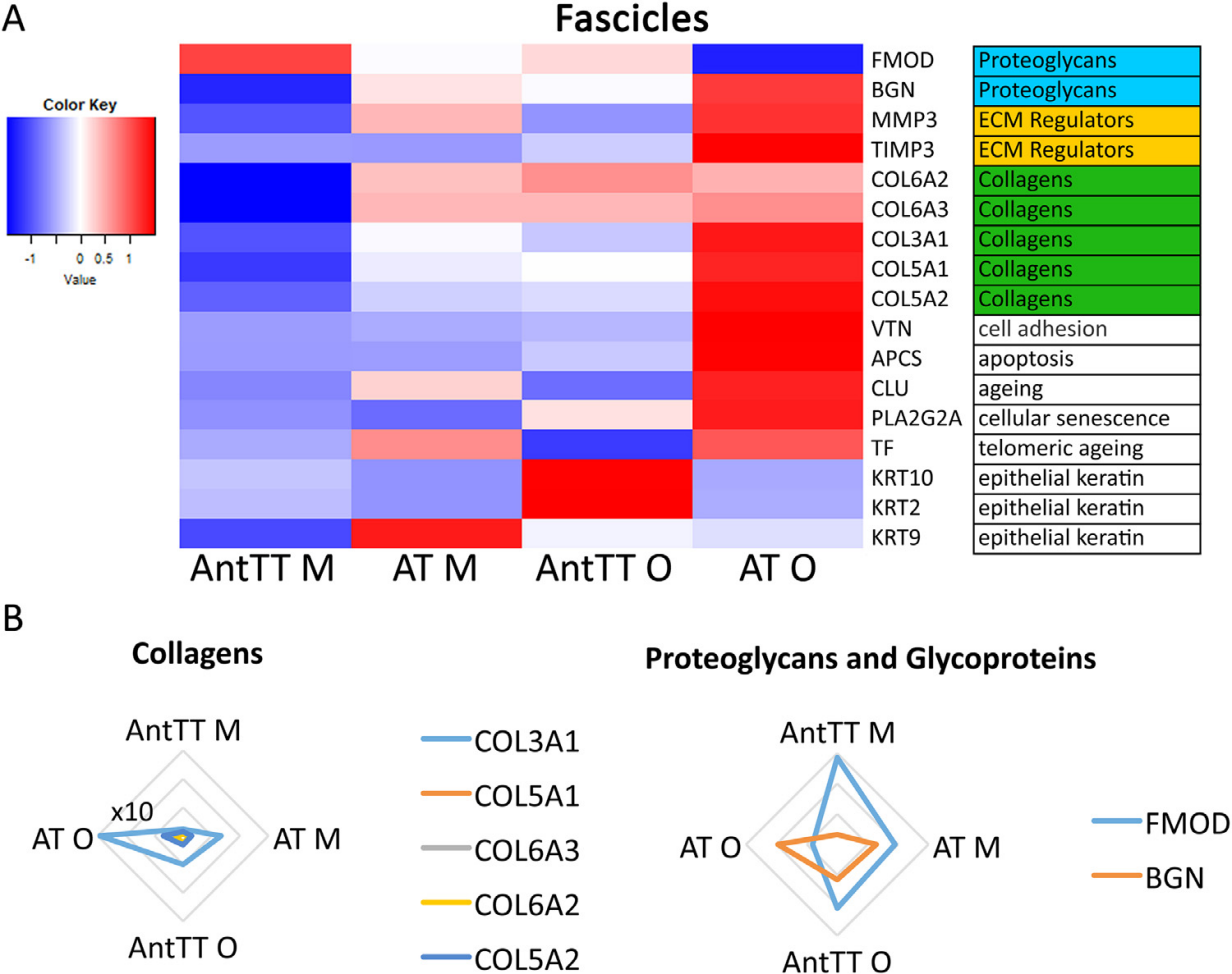
Most changes in fascicle protein abundance are observed in the old Achilles tendon. (A) Heatmap of differentially abundant proteins in the fascicles of middle-aged anterior tibial (AntTT M) and Achilles tendon (AT M), and old anterior tibialis tendon (AntTT O) and Achilles tendon (AT O) (p<0.05, fold change ≥2). Heatmap colour scale ranges from blue to white to red with blue representing lower abundance and red higher abundance. MatrisomeDB was used to assign protein classifications. (B) Radar plots of collagens, proteoglycans and glycoproteins that showed differential abundance with age or tendon type in fascicles (p<0.05, fold change ≥2).

More proteins were identified as differentially abundant in the IFM than in fascicles, with most changes occurring in the old Achilles (Fig. 4 & 5). Many of these proteins were ECM or ECM-associated, with a predominance of proteoglycans and glycoproteins. By contrast, fewer alterations in protein abundance were observed in fascicles, and the majority of those that did change were either collagens, or were not ECM-related. Radar plots indicate that in the IFM, changes in collagen abundance were consistent for different collagen types, whereas differences in proteoglycan/glycoprotein abundance differed with protein type (Fig. 4). A similar pattern was seen for collagens in fascicles as in the IFM, with greater abundance of several collagens in the old Achilles (Fig. 5). A few proteins associated with senescence and ageing were also found more abundantly in the old Achilles tendon, both in the IFM and FM.

#### 3.5.2 Pathway analysis

**Fig. 6:**
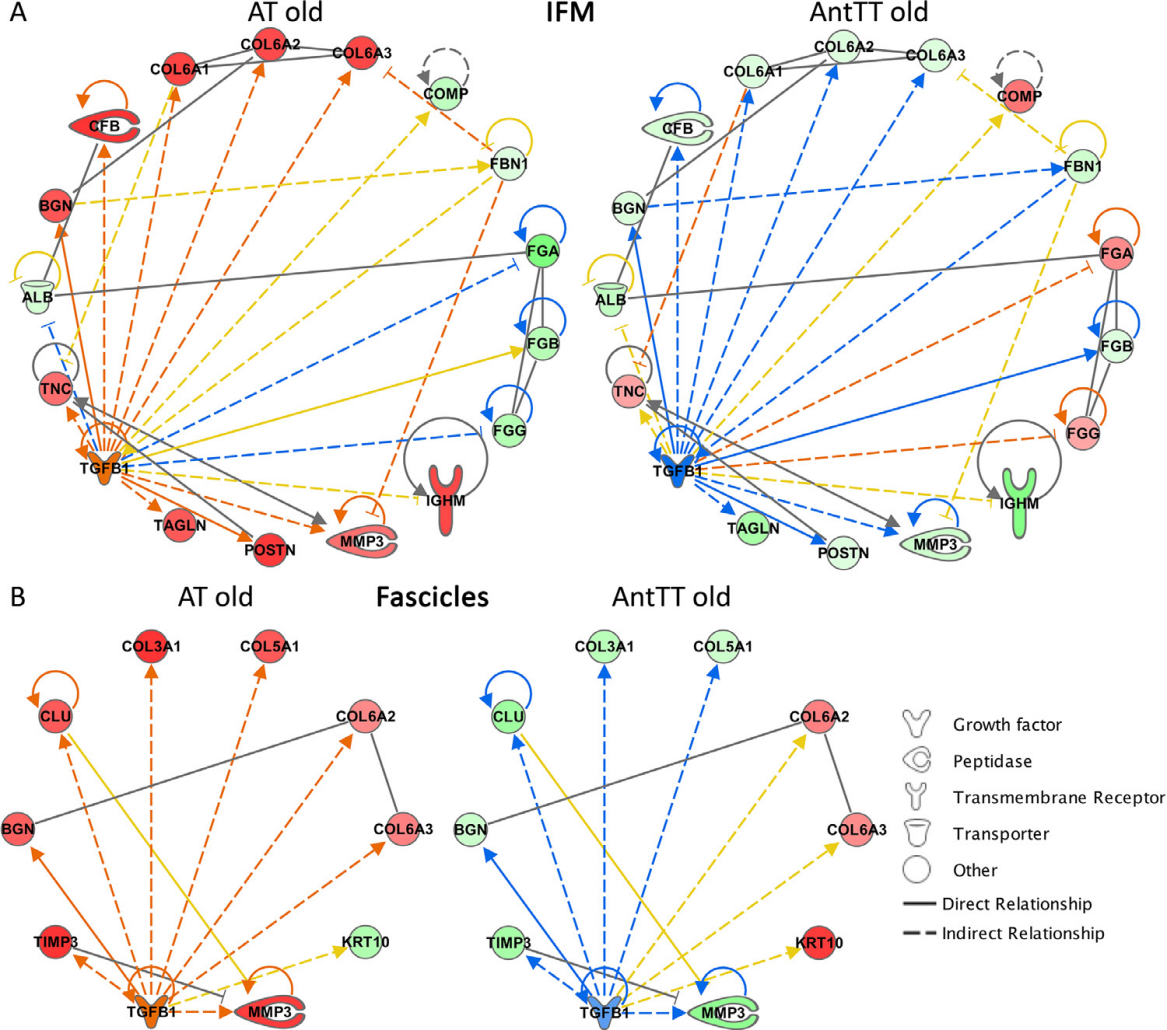
Pathway analysis identified TGF-β1 as an upstream regulator. TGF-β1 is predicted to be activated in the Achilles tendon, but inhibited in the anterior tibialis tendon from aged donors. IPA networks for TGFB1 in the IFM (A) and fascicles (B) of the Achilles tendon and anterior tibialis tendon of old donors. Red nodes: upregulated proteins; green nodes: downregulated proteins; intensity of colour is related to higher fold-change; orange nodes: predicted upregulated proteins in the dataset; blue nodes: predicted downregulated proteins.

Potential upstream regulators were identified using IPA. TGF-β1 was predicted to be activated in the old Achilles, and inhibited in the anterior tibial tendon, in both the IFM and fascicles (Fig. 6).

## 4 Discussion

In this study, we investigated the mechanical and structural specialisations in the human Achilles tendon and established how these altered with ageing. At the fascicle level, there were few differences between tendon types or with ageing. However, in support of our hypothesis, results demonstrate specialisation of the IFM in the Achilles tendon, identifying significantly greater cycles to failure and reduced hysteresis, indicative of more elastic and fatigue resistant behaviour than in the anterior tibial tendon IFM. In contrast to our hypothesis, few changes in mechanical properties were observed with ageing, although there was a trend towards decreased fatigue resistance with ageing in the Achilles IFM only. Proteomic analysis revealed a more complex proteome in the IFM, with age-related alterations in protein abundance predominantly occurring in the Achilles IFM.

Overall, the results we present here are similar to those reported previously in functionally distinct equine tendons, with greater elasticity in energy storing compared to positional tendons and their subunits [12,13]. However, we observed fewer differences between human tendons than seen previously in equine tendon, in which whole tendons and fascicles also show several differences in mechanical properties between tendon types [12,15]. This may simply arise from the lower numbers of available human samples and high sample variability, but may also be associated with the unmatched age ranges of human and equine samples, or with differences in energy storing function, as the Achilles is a less extreme energy store than the highly specialised equine SDFT [2,32].

We were not able to obtain tendons from donors under 30 years old, so we are comparing middle-aged and old, rather than young and old, providing less age contrast than in our previous studies of equine tendon, in which we have demonstrated age-related loss of specialisation within the IFM, and also to a lesser degree within fascicles [27,[33], [34], [35]]. It is possible that specialisation of the Achilles was already diminishing in the middle-aged group, as we observed previously in equine tendon [17], so fewer age-related alterations in mechanical properties were evident. Indeed, it is well established that Achilles tendinopathy is most prevalent in the fourth decade of life [9,36], which may well result from this diminishing specialisation coinciding with continual or increasing usage as individuals take up new sporting activities. While the effect of ageing on energy storage capacity in the human Achilles has not been measured, rodent studies indicate a loss of energy storing capacity in tendon with ageing, which may contribute to the reduction of locomotory efficiency observed with ageing [37].

However, it is also apparent that there are large variations in tendon mechanical properties between individuals, which may mask differences between tendon types or with ageing. The source of this variation is uncertain, but it should be noted that we had a mixed-sex population, and minimal information about the health, exercise or injury status of donors. Tendons with any macroscopic signs of degeneration were excluded from all analyses. However, whilst some donors did have documented diabetes, in others information of any systemic diseases were lacking. While the sex of each donor was known, limited sample numbers means it was not possible to establish if any variability arose from sex-related differences at baseline or with ageing, as previously reported [38]. The presence of sub-tendons within the Achilles arising from the soleus and gastrocnemius muscle bellies further adds to the potential source of variation [39]. While it is possible to estimate IFM shear modulus as approximately 0.6kPa, based on average measurements from previous data, the inability to measure IFM contact area in a non-destructive manner, and subsequently accurately calculate IFM shear modulus for each sample likely increased the variability of these data. Despite these limitations, it is notable that we still identified significant compositional and mechanical differences between energy storing Achilles and positional anterior tibial tendons.

Few studies have investigated the mechanical or structural properties of functionally distinct human tendons and their subunits. Those that have, typically analyse a single tendon type to explore limited mechanical or compositional aspects. The IFM of the human Achilles tendon has received little attention previously, with data supporting the results we present here, demonstrating localisation of lubricin to the IFM [40], and identifying capacity for interfascicular sliding [41]. Indeed, a recent modelling study indicated that sliding of tendon subunits, enabled by IFM, is necessary to accurately predict tendon viscoelasticity and failure [42]. However, in contrast to our previous findings in functionally distinct equine tendons [12,13,17], we did not identify any differences in interfascicular sliding between tendon types or age groups, as measured by IFM extension at the point of maximum stiffness. While the capacity for interfascicular sliding does not appear to differ between tendon types, our results demonstrate enhanced elasticity and fatigue resistance in the Achilles IFM, with the IFM in the Achilles exhibiting 10% less hysteresis, and able to resist approximately a 6-fold greater number of cycles to failure than the IFM in the anterior tibial tendon. Specialisations within the IFM therefore likely contribute to efficient energy storage in the Achilles tendon.

Histological analysis confirmed that the IFM is rich in proteoglycans, particularly lubricin and decorin, and also elastin, as seen previously in tendons from other species [25,43,44]. Mass spectrometry allowed a comprehensive characterisation of the IFM and fascicular proteomes and comparison between tendon types and age groups, revealing a greater complexity in the IFM proteome in both tendon types, with almost double the number of glycoproteins identified in this region compared to the FM, supporting previous findings in equine tendon [14]. The protein profile identified is also similar to that detected previously in the whole Achilles tendon, with many collagens and proteoglycans present [45]. Elastin detection by mass spectrometry requires the inclusion of an elastase digestion step [45] which was not possible with our samples due to the limited volumes collected by laser capture. However, by combining quantitative assays to determine whole tendon elastin content, and immunolocalisation to identify its spatial arrangement, we were able to establish that elastin was localised to the IFM, with a trend towards lower elastin content in the old Achilles compared to the old anterior tibial tendon. Previous research demonstrates a decline in elastin content in the energy storing equine SDFT with ageing [16], and elastin depletion results in alterations in IFM mechanical properties, characterised by small changes in quasi-static mechanical properties, and a large reduction in fatigue resistance [46]. In our samples, a particularly large individual variation in elastin content in the middle-aged Achilles tendon was evident (Supplementary Fig. 1), and due to limited sample availability, we were only able to measure elastin content in a subset of tendons, which may mask a decline in elastin content in this tendon with ageing. Previous studies have demonstrated highly variable energy storage capacity between the Achilles tendons of middle-aged individuals [47], which also indicates significant individual variability in human tendons with ageing.

While no significant changes in tendon or subunit quasi-static mechanics were identified with age, the superior fatigue resistance of the Achilles IFM was lost with ageing. These data suggest some effect of ageing on the Achilles IFM specifically, and indicate that ageing changes may have preserved the overall quasi-static material properties of tendon at the cost of fatigue resistance. Indeed, proteomic analysis revealed that the majority of changes in protein abundance were measured in the IFM from old Achilles tendons relative to the other groups. Many of these changes were ECM-related, suggesting dysregulation of homeostasis which may be responsible for the loss of superior fatigue resistance observed.

This is in contrast to previous transcriptomic analysis of the ageing Achilles, which showed little changes in ECM proteins at the gene level [48]. It may be that the changes in ECM protein abundance we observed are post-transcriptionally regulated, or that separate analysis of IFM and fascicles allows detection of differences that are not apparent when the tendon is analysed as a whole. Indeed, very few proteins changed in abundance in both fascicles and IFM with ageing, instead ageing changes occurred predominantly in the IFM, suggesting differential age-related regulation of protein homeostasis across fascicles and IFM. Of interest, we measured increased abundance of several proteins in the Achilles IFM with ageing that have been associated with fibrosis in connective tissues, including collagen VI and XIV, periostin and tenascin. Collagen VI and XIV are overexpressed in several fibrotic diseases, including pulmonary fibrosis, hepatic fibrosis and adhesive capsulitis, and collagen VI-null mice exhibit improved cardiac structure and function post-myocardial infarction [49], [50], [51], [52], [53]. Indeed, recent studies have implicated collagen VI as a major determinant of fibrosis [54]. Periostin is involved in matrix remodelling across health and disease, and increased levels of periostin are reported in pulmonary and myocardial fibrosis [55], [56], [57]. Tenascin is also increased in fibrotic conditions, and has been reported to drive persistent fibrosis in skin, while its deficiency attenuates fibrosis [58], [59], [60]. The observed increase in proteins in the old Achilles IFM may therefore be linked to fibrotic changes within the IFM leading to decreased fatigue resistance.

In addition, we observed increased MMP-3 in the ageing Achilles. MMP-3 cleaves elastin, and so may contribute to the trend towards decreased elastin content in the Achilles tendon from aged donors. We also identified several proteins associated with cell ageing and senescence, including TAGLN, MAMDC2 and PLA2G2A, that showed increased abundance with ageing in the Achilles, suggesting that cellular senescence may have a role in the age-related changes observed in the Achilles tendon.

TGF-β signalling was predicted to be activated in the Achilles tendon from old donors. TGF-β signalling is essential for tendon development, and is expressed predominantly within the IFM of developing and adult tendons [61], [62], [63]. Indeed, our recent work demonstrates upregulation of TGF-β in the IFM of the energy storing equine SDFT upon commencement of loading during development [29]. However, TGF-β signalling has also been shown to be dysregulated in several age-associated diseases, including atherosclerosis, neurodegenerative diseases and arthritis, and is upregulated in tendon injury [64,65]. In cartilage, TGF-β switches from a protective to a detrimental role with ageing, which is associated with osteoarthritis development [66]. TGF-β has also been identified as a master regulator of fibrosis [67], and it is therefore likely that dysregulation of TGF-β signalling in the old Achilles drives the increase in fibrosis-associated proteins within the IFM. We have previously shown that TGF-β regulation of ECM organisation during development is specific to energy-storing tendons, and is likely to be induced by mechanical loading [29], suggesting that the dysregulation observed during ageing may result from an altered loading environment within the Achilles tendon, either due to changes in activity levels or an age-related deterioration of tendon structure.

## 5 Conclusions

In this study, we demonstrate specialisation of the IFM in the energy storing Achilles tendon, with greater elasticity and fatigue resistance than in the positional anterior tibial tendon. Further, we identify age-related alterations in the IFM proteome of the Achilles tendon which is likely related to the loss of fatigue resistance observed. These changes may contribute to the increased risk of Achilles tendinopathy with ageing, and provide information crucial for developing improved tendinopathy diagnostics, preventative approaches, and IFM-targeted therapeutics.

## CRediT authorship contribution statement

**Dharmesh Patel:** Investigation, Formal analysis, Writing – review & editing. **Danae E. Zamboulis:** Investigation, Formal analysis, Visualization, Writing – review & editing. **Ewa M. Spiesz:** Investigation, Writing – review & editing. **Helen L. Birch:** Conceptualization, Funding acquisition, Writing – review & editing. **Peter D. Clegg:** Conceptualization, Funding acquisition, Supervision, Writing – review & editing. **Chavaunne T. Thorpe:** Conceptualization, Funding acquisition, Writing – original draft. **Hazel R.C. Screen:** Conceptualization, Supervision, Funding acquisition, Writing – review & editing.

## Declaration of Competing Interest

The authors declare that they have no known competing financial interests or personal relationships that could have appeared to influence the work reported in this paper.

## Appendix Supplementary materials

Image, application 1

Image, application 2
